# Anifrolumab for the treatment of refractory chilblain lupus erythematosus

**DOI:** 10.1016/j.jdcr.2023.10.020

**Published:** 2023-11-04

**Authors:** Michael J. Woodbury, Katherine Nabel Smith, Jeffrey S. Smith, Joseph F. Merola

**Affiliations:** a Department of Dermatology, Brigham and Women’s Hospital, Harvard Medical School, Boston, Massachusetts; b Department of Dermatology, UT Southwestern Medical Center, Dallas, Texas

**Keywords:** anifrolumab, chilblain lupus erythematosus, type I interferon

## Introduction

Chilblain lupus erythematosus (CHLE) is a subtype of chronic cutaneous lupus erythematosus (CLE) that manifests often as pruritic and painful erythematous-violaceous papules and plaques at acral regions. The pathogenesis of CHLE is not entirely known; however, the familial TREX1 mutation is implicated, and studies suggest that type I interferon (IFN)-driven signaling contributes to the disease. Additionally, optimal management of recalcitrant disease is unclear.^1^ Here, we describe the workup and successful treatment of 2 patients with recalcitrant CHLE using anifrolumab, a type I IFN receptor antagonist.

## Case descriptions

### Case 1

**Fig 1:**
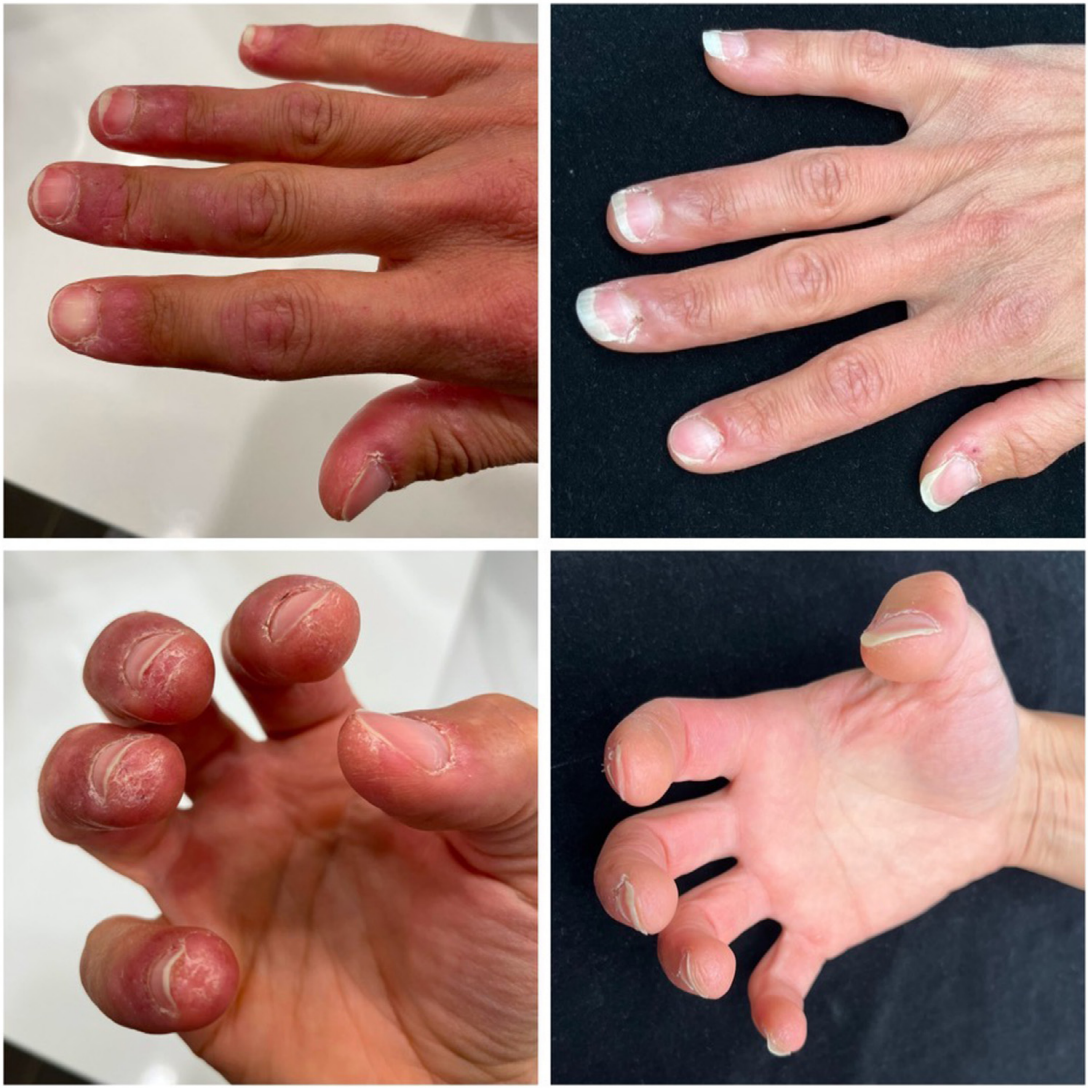
Chilblain lupus erythematosus (CHLE) before and after anifrolumab. These photos represent case 1. The left photos show recalcitrant violaceous scaly plaques on the distal digits consistent with CHLE before treatment with anifrolumab. The right photos show complete resolution of these lesions after 4 monthly infusions of anifrolumab. Photos from case 2 were unable to be obtained.

A 35-year-old female with a history pertinent for autoimmune hepatitis, antiphospholipid antibody syndrome, autoimmune hemolytic anemia, systemic lupus erythematosus (SLE) with discoid lupus erythematosus, and Raynaud disease presented to the clinic for painful lesions on her hands. Her examination was notable for fissuring on bilateral hands as well as prominent nailfold capillary dilation and dropout. Immunologic markers were notable for positive antinuclear antibody, positive anti-Smith, positive antiribonucleoprotein, elevated C-reactive protein, and low complement components 3 and 4. The remaining relevant serologies were unremarkable. A previous skin biopsy of the right ear was consistent with connective tissue disease. In this clinical context, this patient was diagnosed with CHLE. Over the next 5 years, her CHLE was refractory (and she was intolerant) to a number of treatments facing both her Raynaud disease and CHLE lesions, including hydroxychloroquine, chloroquine, methotrexate, azathioprine, pentoxifylline, aspirin, doxazosin, sildenafil, topical ruxolitinib (alternating with clobetasol), belimumab, mycophenolate mofetil, dapsone, tofacitinib (initial improvement for several months but distal digits remained active), rituximab (given for autoimmune hemolytic anemia), lenalidomide, and botulinum toxin injections to the hands. Given the severity of her disease and lack of response to multiple treatments, she was prescribed anifrolumab 300 mg intravenous infusions every 4 weeks in addition to her current existing regimen of mycophenolate mofetil 1 g twice daily, aspirin 81 mg daily, and pentoxifylline 800 mg twice daily. After 4 infusions (12 weeks), the patient reported marked improvement of her CHLE, specifically with regard to her skin healing. She additionally experienced improvement in arthritis symptoms (Fig 1).

### Case 2

A 48-year-old female with a history notable for SLE and Sjogren syndrome presented with painful lesions on her toes. Her clinical examination was notable for erythematous-violaceous plaques with some overlying scale occurring on all toes. Her laboratory work was notable for positive antinuclear antibody, positive double stranded DNA, low complement components 3 and 4, elevated C-reactive protein, positive anti-Ro, and positive anti-La. The remaining relevant serologies were unremarkable. A skin biopsy from 10 years prior of an active lesion on the left arm showed an inflammatory pattern with interface changes and lichenoid infiltrate with scattered dyskeratotic keratinocytes, possibly consistent with CLE. In this clinical context, this patient was diagnosed with CHLE. Over the next 10 years, her CHLE was refractory to prednisone, hydroxychloroquine, chloroquine, methotrexate, pentoxifylline, aspirin, belimumab, mycophenolate mofetil, lenalidomide, and thalidomide. Although thalidomide and lenalidomide were effective, she unfortunately developed intolerable peripheral neuropathy; although it was unclear whether these symptoms were related to lenalidomide, the medication was discontinued given symptom worsening after switching from thalidomide to lenalidomide. Anifrolumab 300 mg intravenous infusions were added every 4 weeks to her existing regimen of aspirin 81 mg daily, chloroquine 250 mg daily, pentoxifylline 400 mg daily, and methotrexate 20 mg weekly. After 2 infusions, the patient reported remarkable improvement in her cutaneous disease, and she remains well-controlled on this regimen 20 months later.

## Discussion

Treatment options for patients with CLE, including CHLE, may be limited by insufficient medication efficacy, comorbidities, or other contraindications. Novel therapeutic options are therefore needed for refractory cases. Our experience with these 2 patients presents evidence that anifrolumab may be an effective option in patients with recalcitrant CHLE.

Anifrolumab is a monoclonal antibody that antagonizes the type I IFN receptor by binding to the IFN-α/β receptor subunit 1. Anifrolumab is Food and Drug Administration-approved for adult patients with moderate-to-severe SLE; however, emerging data suggest it may be a viable option for CLE as well.^2^ In a randomized, double-blind, placebo-controlled study of 305 patients with moderate-to-severe SLE, the percentage of patients with a cutaneous lupus erythematosus disease area and severity index score of ≥10 who improved by ≥50% at week 52 was superior with both anifrolumab doses (300 mg: 63%; 1000 mg: 58%) as compared with placebo (31%).^2^ Additionally, anifrolumab proved efficacious for patients with refractory CLE in multiple case reports^2^, ^3^, ^4^, ^5^, ^6^, ^7^ and in 2 small prospective studies,^8^^,^^9^ one of which had a patient with CHLE. This patient with CHLE showed a clinically meaningful improvement by 3 months, consistent with the patients in our case report. The aforementioned prospective study noted that clinical remission correlated with certain interferon-stimulated genes.^7^ Another study reported histology and immunohistochemical staining of both familial and nonfamilial CHLE and showed that levels of myxovirus resistance protein A, a type I IFN-induced protein, is highly expressed in both forms of CHLE.^10^ These clinical and molecular data therefore support the use of agents that target type I IFN signaling, such as anifrolumab in CHLE and other variants of CLE. These 2 additional patients provide further evidence that anifrolumab may be an effective option for patients with recalcitrant CHLE.

## Conflicts of interest

Dr Merola is a consultant and/or investigator for Merck, AbbVie, Dermavant, Eli Lilly, Novartis, Janssen, UCB, Celgene, Sanofi, Regeneron, Arena, Sun Pharma, Biogen, Pfizer, EMD Serono, Avotres, and Leo Pharma. Dr J.S. Smith is a consultant and/or investigator for Biogen, and thanks the Dermatology Foundation and T32AR007098, Harvard Dermatology Training Grant for support. Author Woodbury and Dr K.N. Smith have no conflicts of interest to declare.
